# Piperine Impedes Biofilm Formation and Hyphal Morphogenesis of *Candida albicans*

**DOI:** 10.3389/fmicb.2020.00756

**Published:** 2020-05-13

**Authors:** Arumugam Priya, Shunmugiah Karutha Pandian

**Affiliations:** Department of Biotechnology, Alagappa University, Karaikudi, India

**Keywords:** piperine, oral candidiasis, antibiofilm, spontaneous resistance, yeast to hyphal transition, hyphal to yeast transition, wrinkle morphology, HBECs

## Abstract

*Candida albicans* is the primary etiological agent associated with the pathogenesis of candidiasis. Unrestricted growth of *C. albicans* in the oral cavity may lead to oral candidiasis, which can progress to systemic infections in worst scenarios. Biofilm of *C. albicans* encompasses yeast and hyphal forms, where hyphal formation and yeast to hyphal morphological transitions are contemplated as the key virulence elements. Current clinical repercussions necessitate the identification of therapeutic agent that can limit the biofilm formation and escalating the susceptibility of *C. albicans* to immune system and conventional antifungals. In the present study, a plant-derived alkaloid molecule, piperine, was investigated for the antibiofilm and antihyphal activities against *C. albicans.* Piperine demonstrated a concentration-dependent antibiofilm activity without exerting negative impact on growth and metabolic activity. Inhibition in the hyphal development was witnessed through confocal laser-scanning microscopy and scanning electron microscopy. Interestingly, piperine displayed a tremendous potential to inhibit the virulence-associated colony morphologies, such as filamentation and wrinkling. Furthermore, piperine regulated morphological transitions between yeast and hyphal forms by inhibiting hyphal extension and swapping hyphal phase to yeast forms yet under filamentation-inducing circumstances. Remarkably, piperine-challenged *C. albicans* exhibited low potential for spontaneous antibiofilm resistance development. In addition, piperine effectively reduced *in vivo* colonization and prolonged survival of *C. albicans*-infected *Caenorhabditis elegans*, thereby expounding the distinct antivirulent potential. Transcriptomic analysis revealed piperine significantly downregulating the expression of several biofilm related and hyphal-specific genes (*ALS3*, *HWP1*, *EFG1*, *CPH1*, etc.). Furthermore, no acute toxicity was observed in the HBECs and nematodes exposed to piperine. Altogether, results from this study reveals the potential of piperine to inhibit biofilm and hyphal morphogenesis, and its *in vivo* efficacy and innocuous nature to HBECs suggests that piperine may be considered as a potential candidate for the treatment of biofilm-associated *C. albicans* infection, especially for oral candidiasis.

## Introduction

Members of *Candida* spp. are commensal inhabitants in human microbiota, which expediate their encounter with host surfaces, such as mucosal linings and abiotic prosthetic biomaterials (Ramage et al., 2005). Undesirable predisposing circumstances such as extremes in age, diminished immune system, diabetes mellitus, HIV/AIDS, and exploitation of broad-spectrum antibiotics pave way for *Candida* spp. to mount pathogenicity that consequently trails to candidiasis progressively (Akpan and Morgan, 2002; Millsop and Fazel, 2016). Among various forms of candidiasis, oral candidiasis is depicted to be the most common human fungal infection, specifically in the early and later life (Abu-Elteen and Abu-Alteen, 1998; Meira et al., 2017; Rath, 2017). *C. albicans* is the predominantly encountered species in oral candidiasis patients, irrespective of the denture status (Muadcheingka and Tantivitayakul, 2015). Commensal *C. albicans* in oral cavity generally causes no harm in the healthy individuals, as its growth is restrained by the immune barriers and other commensal microorganisms in the niche (Kaur et al., 2017). However, when any of these barriers are subsided, overgrowth of *C. albicans* can lead to mild local discomfort and altered taste sensation to severe systemic infections with significant morbidity and mortality (Rajendran et al., 2016).

Pathogenicity of opportunistic *C. albicans* infection is contingent on the virulence attributes, which are provoked for its ability to survive under specific environmental stress. The most-emphasized virulent traits of *C. albicans* include morphological transitions between yeast and filamentous forms, phenotypic switching of white to opaque cells, secretion of proteolytic and lipolytic enzymes, biofilm formation, and expression of host-recognizing proteins (Calderone and Fonzi, 2001; Kumamoto and Vinces, 2005; Schaller et al., 2005).

Among the several virulent features, biofilm formation is imperious for *C. albicans* (Ramage et al., 2001; Lopez-Ribot, 2005). Also, *C. albicans* is the predominant fungal species associated with biofilm formation (Douglas, 2002). Biofilms of *C. albicans* are highly heterogeneous encompassing both cellular (matrix enclosed with microcolonies of yeast and hyphal cells) and noncellular (e.g., cell wall like polysaccharides) components. A well-structured *C. albicans* biofilm can be established on both biotic and abiotic surfaces, and resistance to antifungals by sessile-phase cells are ascertained to escalate with increase in biofilm formation (Chandra et al., 2001; Ganguly and Mitchell, 2011; Tobudic et al., 2012). Consequently, the high degree of biofilm-associated resistance hinders the efficacy of currently available therapies (Mathe and Van Dijck, 2013). Thus, in order to conquer the inabilities of current antifungal therapies for curing candidiasis, alternative therapeutic regimens targeting only the virulence attributes of pathogen will be advantageous (Anibal et al., 2010; Mehra et al., 2012).

From ancient times, medicinal plants have been expended to cure a vast range of human ailments. In last two decades, numerous plant-derived molecules have been explored for the antibiofilm activity as a prophylaxis against the biofilm formation (Abraham et al., 2012; Slobodnikova et al., 2016). Hampering the adherence of organism and allowing it to survive in a planktonic state will eventually increase its susceptibility toward the host immune system and the antibiotic treatment (Nogueira et al., 2017). For oral biofilm infections, some of the antibiofilm agents derived from plant essential oils are systemically being evaluated in clinical trials (Lu et al., 2019). These studies provide a promising perspective regarding the use of plant based antibiofilm compounds for the oral care.

With this milieu, the present study was aimed at evaluating the antibiofilm potential of a plant alkaloid piperine against the sessile state of *C. albicans*, a notorious fungal species in the pathogenesis of oral candidiasis. Piperine is a major alkaloid bioactive component found in the seeds of pepper, which is renowned for imparting pungency to it. In ancient medicine, black pepper was used to treat an array of discomforts and diseases including influenza, rheumatism, migraine, etc. (Chavarria et al., 2016; Gorgani et al., 2017). Piperine was also reported for its numerous therapeutic activities such as antimicrobial, antioxidant, chemoprotective, antiinflammatory, neuroprotective activity, etc. (Mittal and Gupta, 2000; Pradeep and Kuttan, 2002). Thus, in the present study unexplored antibiofilm potential of piperine against *C. albicans* was investigated and found to exhibit a spectacular antibiofilm and antihyphal activity.

## Materials and Methods

### Ethics Statement

In the present study, Human Buccal Epithelial Cells (HBECs) were collected from the healthy individuals by a gentle oral swab in the mucosal surface of cheeks, after obtaining a written informed consent. The experimental protocol and the use of HBECs were assessed and approved by the Institutional Ethical Committee, Alagappa University, Karaikudi (IEC Ref No: IEC/AU/2018/5). All methods were carried out in accordance with relevant guidelines and regulations.

### Fungal Strain, Culture Media and Conditions

In this study, a reference fungal strain *C. albicans* obtained from American Type Culture Collection (ATCC 90028) was used. For maintenance, thawed suspension from glycerol stock (30%) stored at ultra-low deep-freezing condition (−80°C) was streaked on yeast extract peptone dextrose (YEPD) agar plates (1.8%) and incubated at 37°C for 24 h and stored at 4°C for further use. Routine culturing for *in vitro* assays was performed with YEPD broth. *In vitro* assays allied with biofilm formation, and hyphal development was carried out with Spider medium comprising 1% of mannitol, 0.2% of dipotassium hydrogen phosphate, and 1% of nutrient broth. To investigate the effect of piperine on the preformed hyphae of *C. albicans*, RPMI medium (HiMedia, India) was used.

### Phytochemicals

Following phytochemicals were used for screening of potential antibiofilm molecule against *C. albicans*: cineole, 4-isopropyl benzaldehyde, α-pinene, chlorogenic acid, α- terpinene, borneol, octadecene, lupenol, eicosene, ellagic acid, catechin, gallic acid, ferulic acid, limonene, piperine, carvacrol, α-methyl benzylamine, stearic acid, naringin, hesperidin, indole-3-acetic acid, eladic acid, caffeine, methyl palmitate, and 2,4,D-palmitoyl L-ascorbic acid. Piperine (PubChem CID: 638024) was procured from HiMedia, India. Stock solution of piperine was prepared as 50 mg mL^–1^ with methanol and stored at room temperature for further use. To inspect the effect of methanol on viability and biofilm formation, medium containing inoculum and devoid of compound alone was added with methanol during initial screening assays as a vehicle control.

### Screening of Phytochemicals With Potential Antibiofilm Activity Against *C. albicans*

A total of 25 different aforementioned phytochemicals was used for screening of potential antibiofilm molecule against *C. albicans* in a 24-well microtiter plate (MTP) assay using two different growth media, *viz*. YEPD and Spider broth for evaluation of growth and biofilm, respectively. Each well was loaded with 1 mL of respective medium, 1% of overnight *C. albicans* culture as inoculum, and phytochemicals at 500 μg mL^–1^ concentration. The plates were incubated at 37°C for 24 and 48 h for YEPD and Spider medium, respectively. After incubation period, the plates were read at 600 nm to assess the change in cell density. For assessment of biofilm, planktonic cells from Spider-grown cells were decanted and washed with PBS to remove loosely bound cells. Surface attached cells were then stained with 0.4% crystal violet for 10 min. Biofilm cells were finally quantified by addition of 15% glacial acetic acid followed by spectroscopic observation at 570 nm.

### Effect of Piperine on Growth and Biofilm of *C. albicans*

#### Determination of Minimum Inhibitory Concentration (MIC)

Impact of piperine on the survival of *C. albicans* was assessed using microbroth dilution method according to the guidelines of Clinical and Laboratory Standards Institute (Clinical and Laboratory Standards Institute [CLSI], 2008). Briefly, 1% of overnight *C. albicans* culture was used to inoculate 1 mL of YEPD broth. Test groups were supplemented with varying concentrations of piperine, ranging from 2 to 1,024 μg mL^1^. YEPD medium alone served as negative control. The plate was incubated for 24 h at 37°C. Following incubation, cell density was evaluated by measuring absorbance at 600 nm in multifunctional spectrophotometer (Spectra Max 3, Molecular Devices, United States). For visible assessment of the growth effects, 5 μL of cells from each well was spotted on YEPD agar plate and incubated at 37°C for 24 h, and the image was photographed.

#### Metabolic Viability Assay

Metabolic viability of *C. albicans* control and piperine-treated cells was ascertained through a dye-based assay (Repp et al., 2007). Stock solution of 10 mg mL^–1^ of Alamar blue [Resazurin (7-Hydroxy-3H-phenoxazin-3-one 10-oxide)] was prepared in 1 × phosphate buffered saline (PBS) and stored at 4°C until use. Control and piperine-treated *C. albicans* cells were cultured as previously mentioned. Past incubation, cells were harvested by centrifugation at 8,000 rpm for 10 min and washed twice with PBS. Cell pellets resuspended with 1 mL of 1 × PBS were added with 100 μg of Alamar blue and incubated in dark at 37°C for 18–24 h. Alamar blue added to PBS alone served as negative control. Subsequently, samples were centrifuged, and fluorescent intensity of the supernatant was observed at 560 and 590 nm of excitation and emission wavelengths, respectively.

#### Determination of Minimum Biofilm Inhibitory Concentration (MBIC)

The effect of piperine on the biofilm formation of *C. albicans* was performed in 24-well MTP (Muthamil et al., 2018). Briefly, overnight culture of *C. albicans* (1%) was used to inoculate 1 mL of Spider broth in the absence (untreated control) and presence (treated) of test phytochemical piperine with the increasing concentrations, from 2 to 1,024 μg mL^–1^. Appropriate vehicle and negative controls were maintained parallelly. The plates were incubated statically for 48 h at 37°C. Planktonic cells were aspirated and the biofilm cells that are adhered to the surface of wells were rinsed with sterile distilled water to detach loosely bound cells and allowed to air dry. Successively, the wells were stained with 0.4% crystal violet stain for 10 min at room temperature. Surplus stain that are not bound were removed and washed with sterile distilled water. Stain bound to the biofilm cells was resuspended with 1mL of 15% glacial acetic acid. Absorbance was read at 570 nm using multifunctional spectrophotometer. A minimum of 80% inhibition in biofilm formation was considered as MBIC (Subramenium et al., 2018). The percentage of biofilm inhibition was determined by means of the following formula:

Percentageofbiofilminhibition=[(ControlODnm570

-TreatedODn570m)/ControlODn570m]×100

#### Effect of BIC on Growth of *C. albicans*

To assess the effect of piperine (at BIC-32 μg mL^–1^) on growth of *C. albicans*, growth curve analysis was performed in the absence and presence of piperine and amphotericin B (antifungal, positive control for growth inhibition) over a 24-hour period with 1-hour time interval, spectroscopically. Growth curve was plotted as absorbance at 600 nm against time interval.

### *In situ* Visualization of Biofilm Inhibition

For microscopic visualization, *C. albicans* cells in the absence and presence of piperine at BIC and sub-BIC concentrations were allowed to form biofilm for 48 h at 37°C on a 1- × 1-cm glass surface immersed in 1 mL of Spider medium. The glass slide pieces were then washed twice with PBS and air-dried. Based on the microscopic analysis to be performed, slides were further processed accordingly (Prasath et al., 2019).

#### Light Microscopic Analysis

For light microscopic visualization, glass sides were stained with 0.4% crystal violet and incubated for 10 min. Excess stain was removed by washing in distilled water and air dried. Crystal violet stained biofilm cells were visualized under light microscope (Nikon Eclipse 80i, United States) at a magnification of ×400, and the images were documented with the accompanied digital camera.

#### Confocal Laser Scanning Microscopic (CLSM) Analysis

For CLSM, glass slides were stained with 0.1% acridine orange and incubated in dark for 5 min. Slides were washed with distilled water to remove excess stain. Subsequently, the slides were air-dried and viewed under CLSM (LSM 710, Carl Zeiss, Germany).

#### Scanning Electron Microscopic (SEM) Analysis

For SEM analysis, the biofilm cells formed on the glass slides were fixed with 2.5% glutaraldehyde for 1.5 h, followed by dehydration for 5 min with increasing concentrations of ethanol (20, 40, 60, 80, and 100%). Eventually, the slides were air-dried completely and visualized under SEM (Quanta FEG 250, United States).

### Effect of Piperine on Virulence Attributes of *C. albicans*

#### Effect of Piperine on Filamentous Morphology

Effect of piperine on filamentous morphology of *C. albicans* was appraised by the use of Spider medium supplemented with fetal bovine serum (FBS), as described by Muthamil et al. (2018), with minor modifications. Initially, the Spider agar was autoclaved and, when the temperature dampened to 55°C, 5% of FBS and piperine at sub-BICs and BIC were added. Solid agar devoid of compound alone served as control. After proper solidification, 5 μL of 24 h old *C. albicans* culture was spotted on the center of agar surface and the plates were kept for incubation at 37°C for 5 days. The observed filamentous morphology was documented using Gel documentation system (Bio-Rad Laboratories, XR+, United States).

#### Impact on Wrinkle Morphology

Effect of piperine on colony morphology of *C. albicans* was appraised using solid agar medium. Autoclaved YEPD agar medium was added with sub-BICs and BIC of piperine once the temperature dampened below 55°C. YEPD agar plate without piperine was deliberated as control. Later, 5 μL of overnight *C. albicans* culture was spotted at the midpoint of agar surface and incubated at 37°C for 72 h. At the end of incubation period, the colony morphologies were documented using gel documentation system.

#### Effect on Yeast-to-Hyphal Transition

Influence of piperine on the yeast-to-hyphal phase transition of *C. albicans* was reckoned by the method described by Messier et al. (2011), with presumed alterations. Briefly, 1% of overnight culture of *C. albicans* was inoculated in YEPD liquid medium comprising 10% FBS and incubated at 37°C under aerobic conditions with constant shaking at 160 rpm. Piperine in BIC/sub-BIC was added in the treatment groups whereas the similar constitution without piperine was deliberated as control. Yeast cells and hyphal forms were figured and documented by light microscopic observation after 4 h of incubation.

#### Effect on Hyphal-to-Yeast Transition

Efficacy of piperine to switch hyphal form to yeast cells was scrutinized by the procedure illustrated by Vediyappan et al. (2013). *C. albicans* was allowed to form hyphae by incubating in RPMI medium for 4 h at 37°C with gentle shaking. Consequently, after 4 h, piperine at sub-BICs and BIC was added and observed under light microscopy after 2, 4, and 6 h of incubation.

#### Assessment of Spontaneous Resistance Development by *C. albicans* Biofilm Against Piperine

Development of spontaneous resistance to piperine by *C. albicans* biofilm was determined based on the concept described by Min et al. (2017), which was further expounded according to this study. Overnight culture of *C. albicans* was adjusted to 1 × 10^8^ cells and then challenged with varying concentrations of piperine (1 × and 10 × of the sub-BICs and BIC) on YEPD agar plates and incubated at 37°C for 48 h. Prior to documentation, a single colony from each plate (control and piperine challenged) was used to inoculate YEPD liquid medium, which was further streaked on YEPD agar plates and maintained at 4°C for subsequent biofilm assays. Thereafter, MBIC was assessed for all the piperine-challenged *C. albicans* cells, similar to the protocol stated for determination of MBIC.

In addition to this, subsequent passage assay was performed from lowest (8 μg mL^–1^) to highest concentration of piperine (128 μg mL^–1^). Initial passage was commenced with 8 μg mL^–1^ concentration, and for subsequent passages, *C. albicans* was challenged twice with each concentration (8, 16, 32, 64 and 128 μg mL^–1^). A total of 10 passages was performed, and control and treated *C. albicans* from each passage were subjected to following analysis to assess the effect of continuous exposure of piperine on spontaneous resistance development. Initially, control and treated *C. albicans* from each passage were allowed to form biofilm on 24 well-MTP, followed by assessment of biofilm biomass through the crystal violet staining method, as described earlier.

To evaluate the hyphal extension, cells were incubated in hyphal inducing RPMI medium for 4 h and induction of hyphae was assessed microscopically after 1 and 4 h of incubation using Fluorescent microscope (Nikon Eclipse Ts2R, Japan).

In addition to surface attached biofilm and hyphal extension, air-liquid interface biofilm formation was evaluated through flor formation assay. Briefly, control and treated *C. albicans* from each passage was introduced as inoculum in 2 ml YEPD broth and incubated for 24 h. After incubation, the cells were pelleted by centrifugation and washed twice with PBS. Then, 2 mL of flor medium containing yeast nitrate broth with 4% ethanol was added and statically incubated at 30°C for 3 days. The biofilm formation on air-liquid interface was documented after incubation period (Prasath et al., 2019).

#### Disintegration off Mature Biofilm

Biofilm disruptive potential of piperine was evaluated using CLSM, with Live/Dead staining (BacLight, Molecular Probes, United States) (Kiran et al., 2015). *C. albicans* was allowed to form biofilm on 1- × 1-cm glass slide surface for 48 h at 37°C. Spent medium from the wells was decanted and piperine at 2 × BIC was supplemented to the wells added with fresh Spider broth. After 3 h of incubation at 37°C, glass slides were washed twice with PBS and stained with propidium iodide (PI) and SYTO9 (1 μM concentration of each) for 5 min. Unbound stains were excluded by a gentle rinse in PBS, and eventually, biofilm cells were visualized under CLSM.

### Gene Expression Profiling by Real-Time PCR

Total RNA was extracted from *C. albicans* culture grown for 24 h at 37°C with a constant shaking at 160 rpm in the absence and presence of piperine at its BIC (32 μg mL^–1^) by Trizol method of RNA isolation according to the manufacturers’ protocol. Isolated RNA was dissolved in 20 μL of 0.1% diethylpyrocarbonate (DEPC)-treated water and the quality check was performed by agarose gel electrophoresis. Subsequently, RNA samples were reverse-transcribed into cDNA using high-capacity cDNA Reverse Transcription Kit (Applied Biosystems, United States) in the thermal cycler (Eppendorf, Germany).

qPCR reactions were performed in a total volume of 10 μL. The primers for the individual genes were added discretely with SYBR Green Master Mix (Applied Biosystems, United States) reagents at a predefined ratio. Expression profile of candidate genes (list of genes, primer details, and function are provided in Table 1) were analyzed using the thermal cycler (7500 Sequence Detection System). The expression pattern of candidate genes was normalized against β-actin expression (housekeeping gene) and quantified using the ^Δ^ ^Δ^ CT method (Livak and Schmittgen, 2001).

**TABLE 1:** List of genes, their function in pathogenicity of *C. albicans*, and primer sequences used in the study.

S.No	Gene	Function	Primer sequence (5′-3′)		Candida Genome Database Reference number
			Forward	Reverse	
1	*CPH1*	Transcription factor involved in pseudohyphal and hypha formation	TATGACGCTTCTGGGTTTCC	ATCCCATGGCAATTTGTTGT	*C. albicans* CPH1/C1_07370C
2	*CDR2*	ATP-binding cassette (ABC) superfamily, Multidrug transporter	ATGGCTATTGTTGAAACTGTCATTG	CCCTTTACCGAAAACTGGAGTAG	*C. albicans* CDR2/C3_04890W
3	*EFG1*	Transcriptional regulator for switch between white and opaque cells. Required for biofilm formation	GCCTCGAGCACTTCCACTGT	TTTTTTCATCTTCCCACATGGTAGT	*C. albicans* EFG1/CR_07890W
4	*HST7*	Required for opaque mating or white biofilm formation	TCATCAGCTTCTTCTATAC	TATTGAGGAAATGACAGTT	*C. albicans* HST7/CR_03900W
5	*UME6*	Transcriptional regulator of filamentous growth	ACCACCACTACCACCACCAC	TATCCCCATTTCCAAGTCCA	*C. albicans* UME6/C1_06280C
6	*EAP1*	Cell adhesion, filamentation and invasion	TGTGATGGCGGTTCTTGTTC	GGTAGTGACGGTGATGATAGTGACA	*C. albicans* EAP1/C2_09530W
7	*HWP1*	Hyphal development, biofilm formation	GCTCCTGCTCCTGAAATGAC	CTGGAGCAATTGGTGAGGTT	*C. albicans* HWP1/C4_03570W
8	*RAS1*	Cell adhesion, filamentous growth, hyphal induction	CCCAACTATTGAGGATTCTTATCGTAAA	TCTCATGGCCAGATATTCTTCTTG	*C. albicans* RAS1/C2_10210C
9	*ALS3*	Adhesion, Agglutinin like protein	CAACTTGGGTTATTGAAACAAAAACA	AGAAACAGAAACCCAAGAACAACC	*C. albicans* ALS3/CR_07070C
10	*TUP1*	Transcriptional repressor, represses filamentation, regulates switching	CTTGGAGTTGGCCCATAGAA	TGGTGCCACAATCTGTTGTT	*C. albicans* TUP1/C1_00060W
11	*ALS1*	Cell surface adhesin	CCTATCTGACTAAGACTGCACC	ACAGTTGGATTTGGCAGTGGA	*C. albicans* ALS1/C6_03700W
12	*CDR4*	Putative ABC transporter superfamily	TACGGTGCTCCAAAATGTCC	TGGGCAGCAATAAATGTAATCC	*C. albicans* CDR4/C1_08070W
13	*ECE1*	Hyphal specific protein, Candidalysin	CCAGAAATTGTTGCTCGTGTTGCCA	TCCAGGACGCCATCAAAAACGTTAG	*C. albicans* ECE1/C4_03470C

### Effect of Piperine on *C. elegans* Survival and *in vivo* Biofilm of *C. albicans*

Toxicity of piperine and its impact on the biofilm formation of *C. albicans in vivo* condition was reviewed using a simple eukaryotic model organism *C. elegans*, which is often expended in toxicological investigation. Wild-type nematode (N_2_) procured from *C. elegans* Genome Center (CGC) was used in this study. For all the evaluation, a synchronized population of nematodes (young adult) was obtained through bleaching. For assessment of toxicity and rescue, approximately 10 worms were transferred to 24-well MTP containing M9 buffer. *E. coli* OP50 (1 × 10^3^ cells) was used as a food source for the worms. Worms were segregated into four groups: (i) control group (*E. coli* OP50 alone); (ii) vehicle control containing methanol in addition to food source; (iii) piperine at various concentrations (16, 32, 64, 128, and 256 μg mL^–1^); and (iv) *C. albicans* cells in the absence and presence of piperine at 16, 32, and 64 μg mL^–1^, respectively. Survival rate of worms were monitored for every 24 h for 9 days using a stereo microscope (Nikon SZ-1000, Japan). For *in vivo* colonization assessment, worms were crushed in saline and plated on candida differential agar. Internal colonization was also observed under CLSM (Swetha et al., 2019; Valliammai et al., 2019).

### Effect of Piperine on Human Buccal Epithelial Cell (HBEC)

As this study is intended to evaluate the therapeutic potential of *C. albicans* for its application in oral candidiasis, safety evaluation was further evaluated with the HBEC. HBECs were collected from the healthy individuals with suitable oral hygiene by gently rubbing a sterile swab on the mucosal surface of the cheeks. Subsequently, the sterile swabs were suspended in sterile PBS and used immediately. The suspended HBECs were further centrifuged, and the pellets were washed thrice with sterile PBS (Souza et al., 2018). A suspension with 5.0 × 10^5^ cells ml^–1^ was prepared by counting the cell suspension through Automatic cell counter (Countess II FL, Invitrogen, United States). HBECs were then incubated with different concentrations of piperine (8, 16, 32, 64, and 128 μg ml^–1^) and incubated for 20 min at 37°C. Hydrogen peroxide was used as positive control. After 20 min of incubation, the cells were stained with crystal violet and visualized under microscope (Nikon Eclipse Ts2R, Japan) to assess morphological variations due to effect of the compound.

### Statistical Analysis

All the experiments were carried out in three biological replicates with at least two technical replicates and values are presented as mean ± standard deviation (SD). To analyze the significant difference between the values of control and treated samples, one-way analysis of variance (ANOVA) and Duncan’s *post hoc* test was performed with the significant *p*-value of <0.05 by the SPSS statistical software package version 17.0 (Chicago, IL, United States).

## Results

### Screening of Phytochemicals With Potential Antibiofilm Activity Against *C. albicans*

**FIGURE 1:**
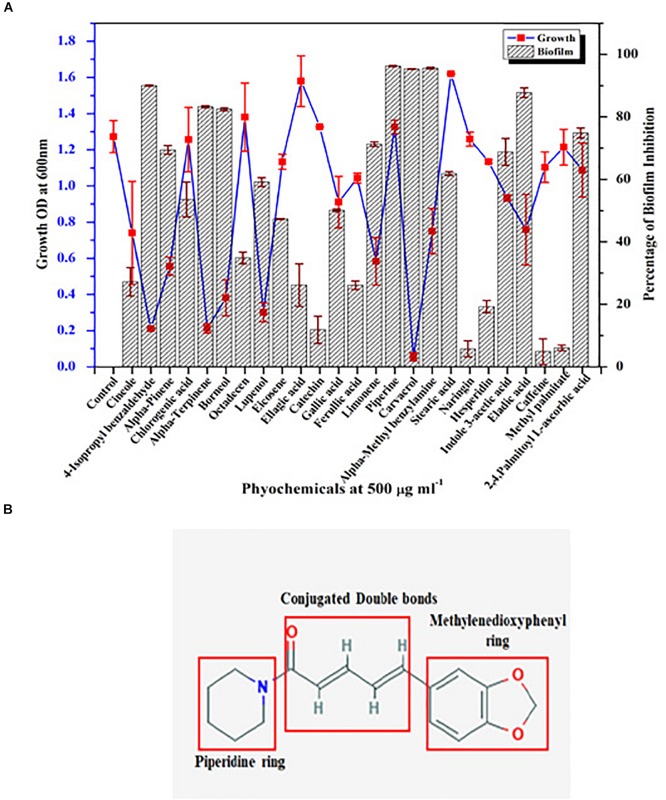
Screening of phytochemical with potential antibiofilm activity against *C. albicans*. **(A)** Screening of various phytochemicals for antibiofilm activity against *C. albicans* through assessment of growth and biofilm inhibitory efficiency. Error bars represent standard deviations from the mean (*n* = 3). **(B)** Chemical structure of piperine, the potential antibiofilm molecule found through screening of various phytochemicals. The three major components in the chemical structure of piperine were boxed within red colored square *viz*. piperidine ring, conjugated double bonds, and methylenedioxyphenyl ring.

Growth and biofilm inhibitory potential of 25 different phytochemicals were evaluated against *C. albicans.* From Figure 1A, in comparison with the effect of phytochemicals on growth and biofilm, piperine was found to be the potential antibiofilm agent against *C. albicans*, as it inhibited >90% of biofilm formation without affecting the growth. All other phytochemicals were found to be either growth inhibitory with increased antibiofilm activity (if growth was found to be inhibited, apparently biofilm will not be formed) (e.g., α-terpinene, borneol) or non-growth inhibitory with no antibiofilm activity (e.g., naringin, hesperidin). Thus, from screening 25 different phytochemicals, piperine was found to be the only compound that had biofilm inhibitory potential without exerting negative impact on growth. Henceforth, piperine was chosen to study its antibiofilm efficacy and influence on other virulence attributes of *C. albicans.*

### Antibiofilm Potential of Piperine With No Significant Impact on Growth and Metabolic Viability of *C. albicans*

**FIGURE 2:**
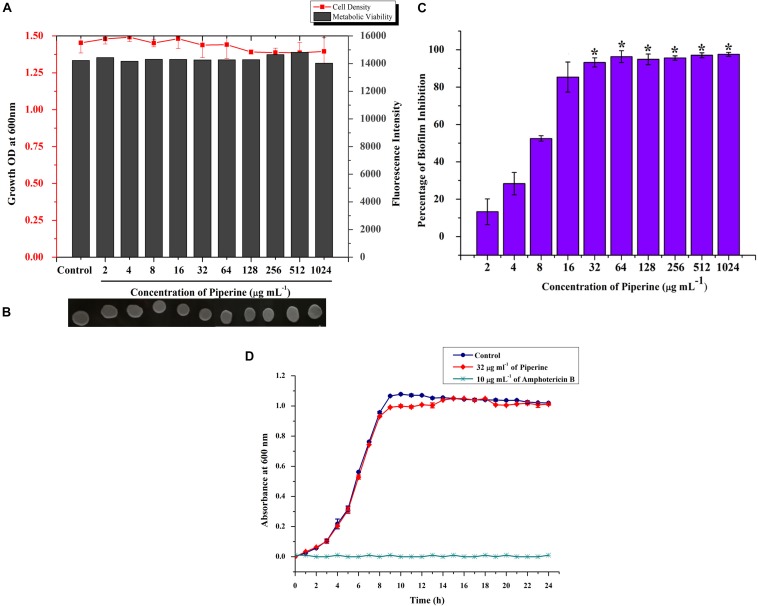
Effect of piperine on growth, metabolic viability, and biofilm formation of *C. albicans.* **(A)** Measurement of cell density and metabolic viability of *C. albicans* in the absence and presence of piperine treatment. **(B)** Spot assay for validation of non-growth inhibitory effect of piperine. **(C)** Effect of piperine on biofilm formation of *C. albicans.* Growth and viability were not found to be significantly altered by piperine whereas biofilm formation was hindered by >90% by the action of 32 μg mL^– 1^ of piperine. Error bars represent standard deviations from the mean (*n* = 3) and *p < 0.05. **(D)** Growth curve of *C. albicans* in the absence and presence of piperine at BIC. Amphotericin B was used as a positive control.

Initially, the impact of piperine on growth of *C. albicans* was investigated by determination of MIC. Primarily, result revealed that the growth of *C. albicans* was not significantly influenced by piperine, even at the highest tested concentration (1,024 μg ml^–1^). For substantiation of non-antifungal effect of piperine, the metabolic viability of *C. albicans* in the absence and presence of piperine was appraised by Alamar Blue assay. On visual observation, the resazurin dye (purple color) was found to be reduced to resorufin, a pink-colored fluorescent dye that evidenced the active cellular metabolism. Furthermore, the fluorescent intensity was found to be unchanged between control and piperine-treated *C. albicans*. For further validation, spot assay confirmed that piperine treatment did not affect the cell growth in comparison with the control. Distinctly, these results depicted that piperine does not exert negative impact on the growth and metabolic viability of *C. albicans* (Figures 2A,B).

Subsequently, evaluation of antibiofilm potential of piperine divulged the dose-dependent decrease in biofilm biomass of *C. albicans.* At 32 μg ml^–1^ concentration, a maximum of 93% of biofilm was found to be inhibited by piperine. Successive concentrations above 32 μg ml^–1^ did not display any significant increase in antibiofilm activity. Hence, 32 μg ml^–1^ was considered as BIC, and further assays were carried out with BIC and sub BICs (Figure 2C).

Further, no significant change in the growth was observed between the growth of *C. albicans* in the absence and presence of 32 μg ml^–1^ of piperine when compared to Amphotericin B, which completely inhibited the growth of *C. albicans* at 10 μg mL^–1^ (Figure 2D).

### Piperine Deteriorates Surface Adherence and Hyphal Development in *C. albicans*

**FIGURE 3:**
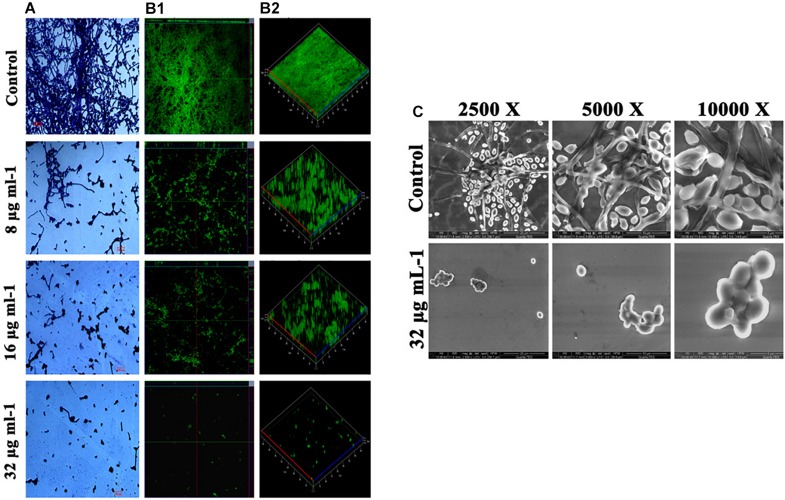
Deterioration in the *C. albicans* surface adherence and hyphal development by piperine. **(A)** Light microscopic micrographs depicting decrease in the biofilm formation and hyphal development. **(B-1,B-2)** Biofilm architecture of *C. albicans* in ortho and 3D view, respectively. **(C)** SEM micrographs displaying the biofilm network of control and piperine-treated *C. albicans* under different magnifications.

Effect of piperine on biofilm architecture of *C. albicans* was perceived through microscopic observations. Light and CLSM micrographs disclosed the well-structured biofilm matrix with yeast and hyphal cells in control, whereas tremendously inhibited microcolony formation and hyphal network in a dose-dependent manner was seen in piperine treatment. In sub-BICs of piperine (16 and 8 μg mL^–1^), shorter hyphal protrusions with scattered yeast cells were observed (Figures 3A,B).

Furthermore, SEM micrographs revealed similar discrepancy in the biofilm architecture of control and piperine-treated *C. albicans.* At BIC, hyphal protrusions and yeast to hyphal transitions were completely suppressed, and few yeast cells were found to be scarcely dispersed, whereas control *C. albicans* displayed a structurally complex and dynamic biofilm network encompassing cells with yeast and hyphal morphologies (Figure 3C).

### Piperine Diminishes the Virulence Traits of *C. albicans*

Various virulence attributes of *C. albicans* was found to be impaired by the ability of piperine.

#### Filamentous Morphology

**FIGURE 4:**
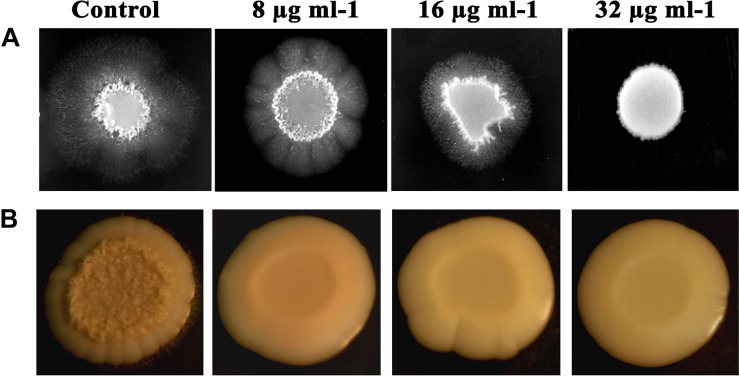
Piperine impeding the primary virulence attributes of *C. albicans.* **(A)** Filamentous development of *C. albicans* under the impact of piperine. **(B)** Wrinkle colony morphology.

Filamentous morphology, especially in hyphal forms of *C. albicans*, contributes to the augmented virulence and pathogenicity, owing to its potential to breach and invade the host cells and confront immune attack. *C. albicans* treated with piperine at BIC was found to be devoid of filamentous growth. Complete suppression of filament development in *C. albicans* indicates the attenuated pathogenicity. Reduction in filamentation was observed in sub-BIC’s of piperine (16 and 8 μg mL^–1^) (Figure 4A).

#### Wrinkled Colony Morphology

Different from the smooth and flat colonies, wrinkled colonies of *C. albicans* facilitate improved access to oxygen, which in turn augments the hyphal development. Supplementing piperine to *C. albicans* culture resulted in the disappearance of wrinkle phenotype, compared to the undulating filaments in untreated control. Even at the lowest sub-BIC (8 μg mL^–1^), no wrinkling was observed which describes the potential antivirulent efficacy of piperine (Figure 4B).

#### Yeast to Hyphal Transition

**FIGURE 5:**
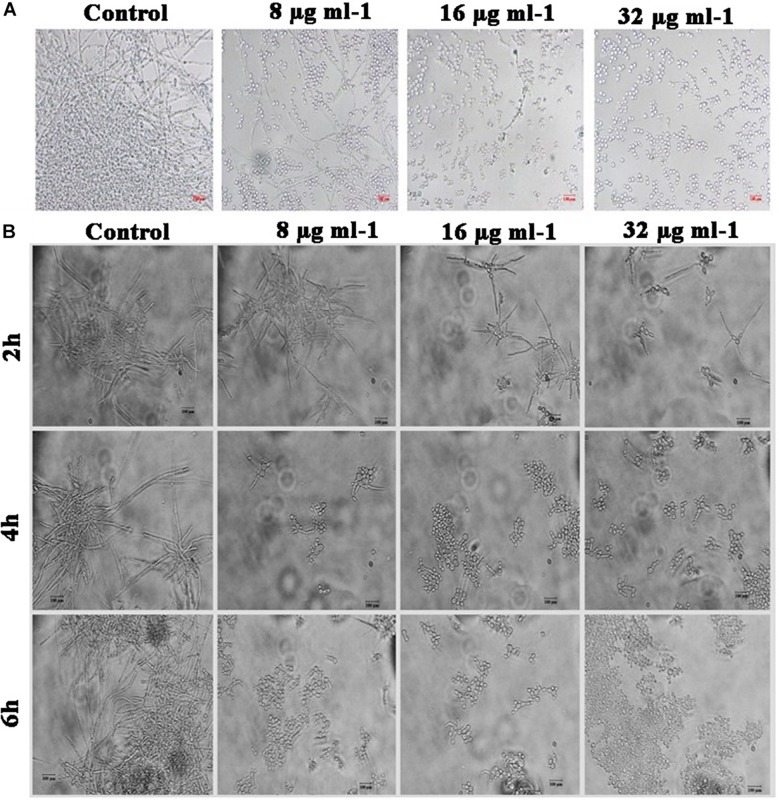
Effect of piperine on phenotypic switching. **(A)** Control in the morphological transition from yeast to hyphal form. **(B)** Transition of preformed hyphal cells to yeast forms in time course (2, 4, and 6 h).

It is postulated that yeast to hyphal transition is one of the prerequisites for the biofilm formation by *C. albicans.* Under hyphal inducing condition (supplemented with FBS), yeast to hyphal transition in piperine-treated and untreated *C. albicans* was microscopically observed. A highly entangled hyphal network with intruded yeast cell aggregates was witnessed in the control, whereas dispersed yeast cells with much-reduced aggregates were spotted in the piperine-treated *C. albicans* culture at its BIC. Dose-dependent response in yeast to hyphal transition was also evidenced. Furthermore, these results were in line with the antibiofilm and hyphal inhibition activities (Figure 5A).

#### Hyphal to Yeast Transition

In addition to restricting yeast to hyphal transition, competency of piperine to switch preformed hyphal cells to yeast state was evaluated under hyphal inducing condition (supplementation of RPMI medium). Preformed hyphal cells incubated with different concentrations of piperine were routinely monitored under light microscope and figured that after 6 h, over 90% of cells were swapped to yeast forms in BIC of piperine. Hyphal to yeast transition was observed in a dose- and time-dependent fashion (Figure 5B).

### Diminished Potential for Spontaneous Resistance Development Against Piperine by *C. albicans*

**FIGURE 6:**
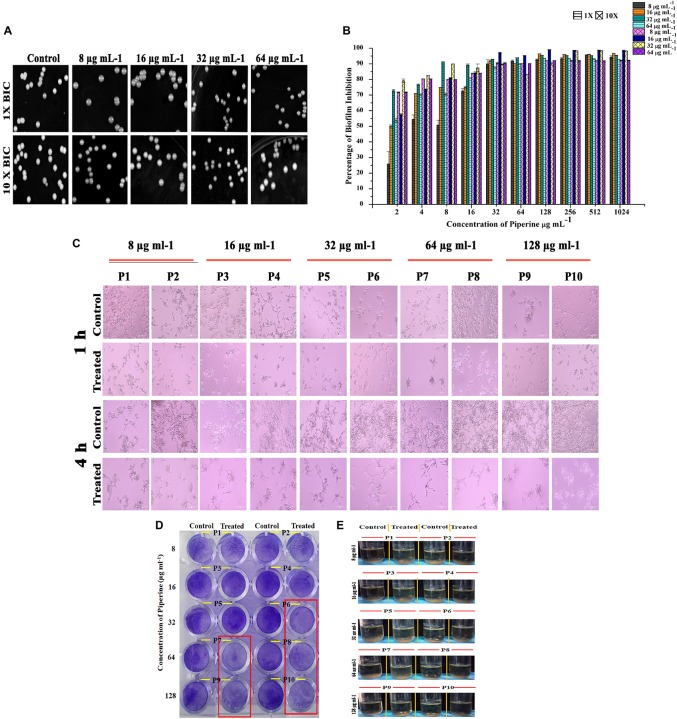
Effect of piperine on spontaneous antibiofilm resistance development **(A)** Growth of *C. albicans* in piperine challenged plates. Top panel displays the *C. albicans* cells grown in the presence of 1 × of 0.25, 0.5, 1, and 2 × BIC. Bottom panel shows *C. albicans* grown under 10 × concentration of 0.25, 0.5, 1, and 2 × BIC. **(B)** Impairment in biofilm formation of piperine challenged *C. albicans* evidencing low potential for spontaneous antibiofilm resistance development. Error bars represent standard deviations from the mean (*n* = 3). **(C–E)** Effect of subsequent passage of *C. albicans* with piperine from lower to higher concentration on **(C)** hyphal extension, **(D)** biofilm formation, and **(E)** flor formation (air–liquid interface biofilm formation).

Resistance development contributes to the recalcitrant and recurrent biofilm formation. Henceforth, the spontaneous antibiofilm resistance to piperine by *C. albicans* was evaluated. *C. albicans* cultured in piperine-challenged (1× and 10× concentrations of 0.25, 0.5, 1, and 2 BIC) environment was assessed for the biofilm forming potential. Primarily, no reduction in number of colonies was observed in control and piperine-challenged plates. Wrinkled morphology was observed in all the colonies of control plates, whereas smooth and flat colonies were observed in the piperine-challenged plates (Figure 6A). This result was in line with the potential to suppress wrinkled colony morphology. In addition, this result also demonstrates the consistent antivirulent potential of piperine. Piperine-challenged *C. albicans* was found to form slightly diminished biofilm which makes it more vulnerable to piperine in consequential supplement. Thus, for piperine challenged *C. albicans*, MBIC in subsequent treatment dropped below 32 μg mL^–1^ (Figure 6B).

A resistance assay carried out by passaging *C. albicans* cells from a lower (8 μg mL^–1^) to a higher concentration (128 μg mL^–1^) of piperine was evaluated for resistance development if any by various assays. Biofilm biomass of *C. albicans* grown in the presence of 32 μg/mL and greater concentration of piperine was found to be reduced (Figure 6D). In addition to this, the habituated cells were grown under hyphal inducing condition (RPMI medium) and found that piperine controlled the extensive hyphal network formation even in the successive passages (Figure 6C).

In addition to the biofilm forming ability and hyphal extension activity, biofilm formed in the air liquid interface was also found to be reduced in the passages continuously exposed to lower to higher concentration of piperine (Figure 6E).

All these results substantiate that piperine does not induce spontaneous or adaptive antibiofilm resistance development in *C. albicans.*

### Dispersal of Mature *C. albicans* Biofilm by Piperine

**FIGURE 7:**
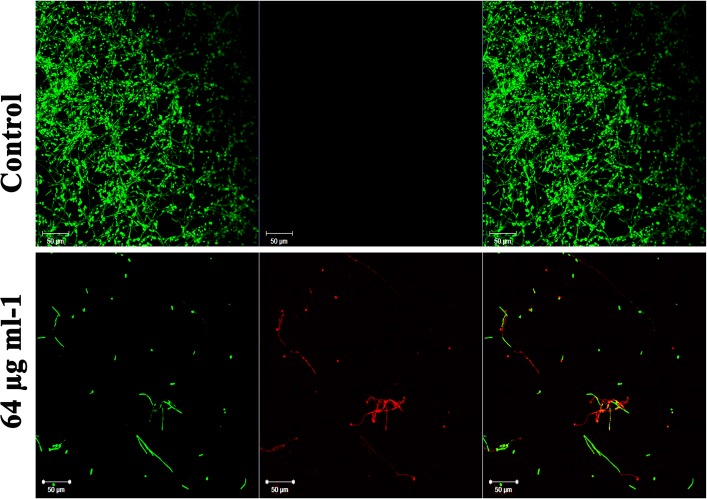
Influence of piperine on disruption of preformed mature biofilm of *C. albicans.* Piperine effectively dislodged biofilm architecture of *C. albicans* at 64 μg mL^–1^ concentration (2 × BIC), leaving behind few hyphal protrusions.

In addition to the antibiofilm potential, the ability of piperine to disrupt the preformed biofilm was perceived through CLSM. Piperine at 2 × BIC effectively dislodged a massive part of the biofilm architecture with few hyphal protrusions left behind. Among them, a scarce population was found to be deceased. This result expounded the mature biofilm-disruptive potential of piperine, which makes it more appropriate for not only the prevention but also for the treatment of established biofilm infection (Figure 7).

### Differential Gene Expressions by Piperine

**FIGURE 8:**
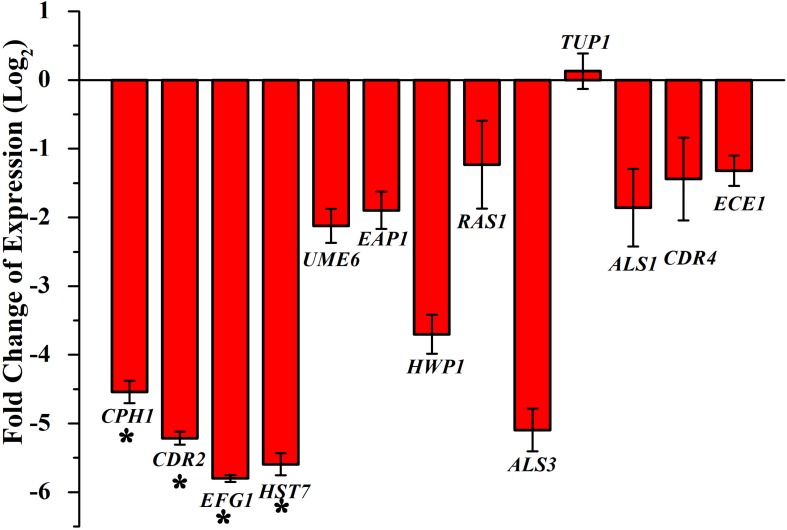
Differential regulation of genes involved in biofilm and hyphal development of *C. albicans* by piperine at BIC (32 μg mL^– 1^). Filamentation-promoting genes, such as *CPH1*, *UME6*, and *EFG1*; hyphal growth-extending genes, such as *EAP1*, *HWP1*, *HST7*, *RAS1*, and *ECE1*; adhesion-supporting *ALS3* and *ALS1*; and drug resistance genes *CDR4* and *CDR2* are significantly downregulated by piperine. *tup1*, transcriptional repressor for the filamentation and hyphae-inducing genes, is upregulated. Error bars represent standard deviations from the mean (*n* = 3). *p < 0.05.

Transcriptional level of hyphal-specific and biofilm-related genes in *C. albicans* in the absence and presence of piperine was quantified by real time PCR. Piperine at BIC significantly downregulated the expression of transcriptional regulators of filamentous growth, such as *CPH1*, *UME6*, and *EFG1*, and hyphal specific genes, such as *EAP1*, *HWP1*, *HST7*, *RAS1*, *ECE1*, and biofilm-related genes such as *ALS3* and *ALS1*. In addition to this, candida drug resistance gene *CDR4* and *CDR2* was found to be significantly downregulated. Furthermore, a transcriptional repressor *TUP1*, which represses the genes responsible for initiating filamentous growth, was found to be upregulated. Altogether, the real-time PCR results substantiated the antibiofilm and antihyphal potential of piperine through significant alteration in the expression of hyphae and biofilm specific genes (Figure 8). Interestingly, the drug resistance was also found to notably downregulated, which could also be correlated with decreased spontaneous resistance development.

### Piperine With No Lethal Effect Diminished *in vivo* Colonization of *C. albicans* in *C. elegans*

**FIGURE 9:**
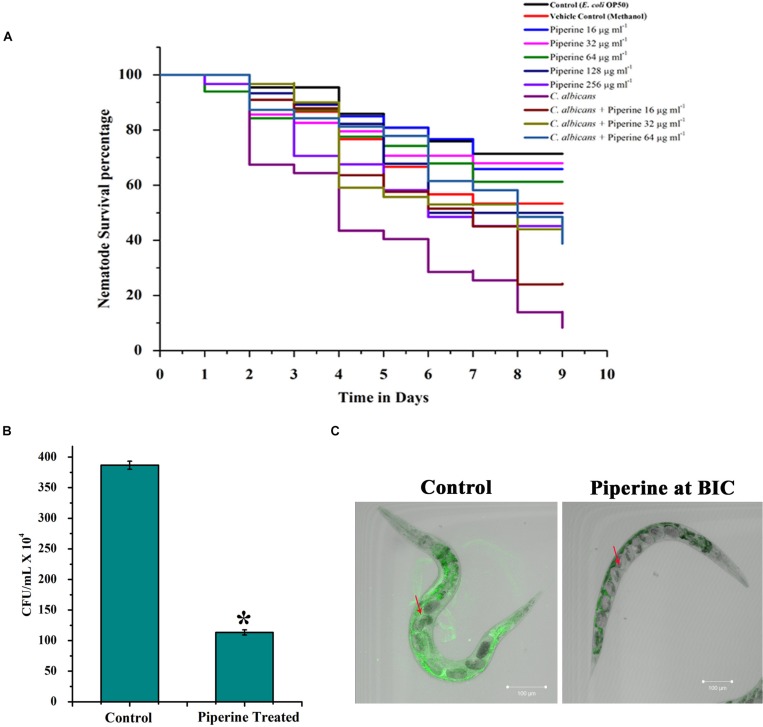
*In vivo* efficacy of piperine on survival and rescue of *C. elegans* from *C. albicans* infection. **(A)** Toxicity effect of piperine and the potential for rescuing *C. elegans* from *C. albicans* infection. **(B)** Effect of piperine on *in vivo* colonization of *C. albicans* in *C. elegans.* Number of *C. albicans* colonized on *C. elegans* was assessed by cfu and **(C)** CLSM micrographs showing reduced colonization of *C. albicans* inside the nematode. The arrow mark in the left panel (Control) shows the deformities and internal hatching of eggs due to the pathogenicity of *C. albicans.* Red arrow in the right panel (treated) denotes the healthy eggs in *C. elegans* rescued from *C. albicans* infection by piperine at 32 μg mL^– 1^ concentration. Error bars represent standard deviations from the mean (*n* = 3). *p < 0.05.

*In vivo* toxic effect, rescuing potential from *C. albicans* infection and effect on *in vivo* antibiofilm potential of piperine was appraised using the *in vivo* model organism *C. elegans.* Toxic effect and potential antiinfective efficacy of piperine against *C. albicans* infection was evaluated by performing *C. albicans*–*C. elegans* liquid infection assay and assessed through survival assay, which revealed that piperine at sub-BIC, BIC, and 2 × BIC does not hold any *in vivo* toxicity. Whereas 4 × and 8 × BIC was found to slightly inhibit the survival of *C. elegans.* The survival of worms exposed to the vehicle control–methanol was also found be decreased similar to the range of 4 × and 8 × BIC-treated worms. Hence, at 4 × and 8 × BIC, it is not predictable to assess where the decreased survival was due to the compound or the vehicle control (Figure 9A). From the *C. albicans*–*C. elegans* liquid infection assay, it is evident that piperine rescued *C. elegans* from *C. albicans* infection. Decreased survival was observed in the *C. albicans*-infected worms, whereas piperine supplementation helped *C. elegans* to combat *C. albicans* infections in a concentration-dependent manner (Figure 9A). This was further reflected in the diminished internal colonization of *C. albicans.* The control worms had about 3.8 × 10^6^ cells whereas piperine treatment reduced the colonization and only 1.13 × 10^5^ *C. albicans* cells were observed (Figure 9B). This was further substantiated through CLSM, wherein the colonization was measured through fluorescent intensity. Interestingly, CLSM micrographs showcased the internal abnormalities in *C. albicans*-infected worms. Deformities in eggs and internal hatching were observed in the infected worms while piperine prevented all these abnormalities in treated group (Figure 9C). Concomitantly, the result pronounces piperine to be an ideal antibiofilm and antiinfective agent as it hinders not only the *in vitro* but also the *in vivo* colonization of *C. albicans.*

### Piperine Does Not Affect the HBECs

**FIGURE 10:**
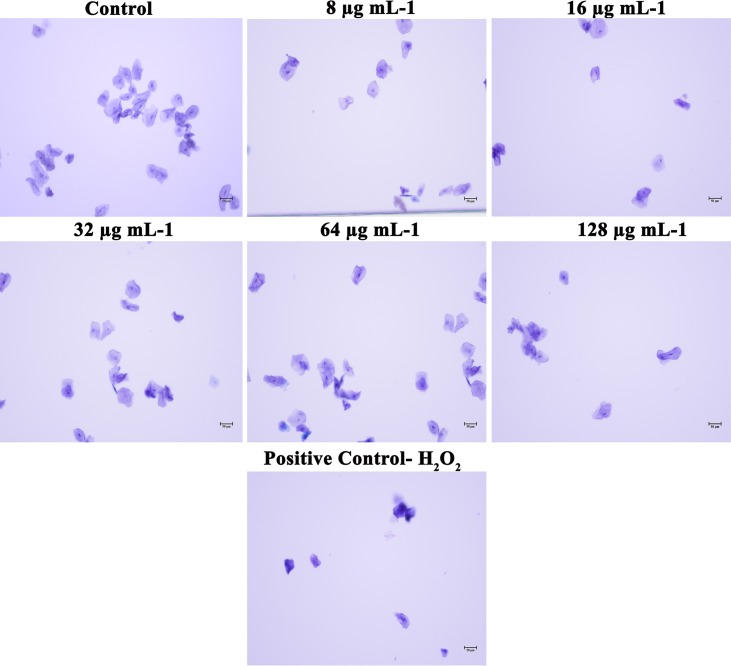
Effect of piperine on HBECs. HBECs treated with 8, 16, 32, 64 and 128 μg mL^– 1^ of piperine were found to be health and normal, as the control cells whereas HBECs treated with H_2_O_2_ were found to be completely distorted.

In addition to the effect of piperine on *in vivo* toxicity and colonization on *C. elegans* animal model, HBECs were used to assess the safety of using piperine for treatment of oral candidiasis. From the microscopic analysis, HBECs were found to be healthy and normal in piperine treatment at 8 to 128 μg mL^–1^ concentration, in comparison to the control cells. The positive control treated with H_2_O_2_ cells were found to be completely distorted. From this result, it can be concluded that piperine treatment is not lethal to the HBECs and found to be safe (Figure 10).

## Discussion

Amid the diverse fungal species, *C. albicans* stands as the predominant organism associated with the biofilm formation, which is one of the major virulence traits devoted to its versatile pathogenicity. The sessile state of *C. albicans* embraces important clinical repercussions, owing to the increased drug resistance and recalcitrant nature and besides more significant morphological diversification and phenotype switching (Ramage et al., 2005). The proficiency of *C. albicans* to establish biofilm on both biotic (epithelial and mucosal membranes) and abiotic surfaces (prosthetics) often leads to candidiasis (Trindade et al., 2015). Since biofilm cells are more resistant to antibiotic therapy, preventing or disintegrating the biofilm formation is postulated to be an effectual therapeutic regimen. Based on this milieu, in order to attenuate the biofilm formation and virulence traits of *C. albicans*, with decreased avenue for resistance development, this study demonstrated the use of a plant alkaloid, piperine, as an effective antibiofilm and antihyphal molecule against *C. albicans* biofilm infection.

Antibiofilm molecule does not affect the survival of an organism and is thus contemplated to have trivial probability for resistance development (Rabin et al., 2015). Piperine markedly reduced the biofilm formation of *C. albicans* at the concentration of 32 μg mL^–1^, without influencing the normal cellular and metabolic viability. Thus, piperine is considered as an ideal antibiofilm agent. Further, microscopic analysis demonstrated that piperine was effective in inhibiting adherence of *C. albicans* to surface and restricted the hyphal development in a dose-dependent manner. Morphological transition of *C. albicans* between yeast and filamentation forms contributes greatly to the pathogenicity. Both the morphological forms are critical to the virulent characteristics of *C. albicans* and exhibit distinct functions at different stages of disease progression, which includes adhesion, invasion and damage to the host tissue, dissemination of the infection, and circumvention from immune evasion and host response (Jacobsen et al., 2012). Notably, invasion and host tissue damage are dependent on the filamentous morphology (Vila et al., 2017). Thus, targeting the dimorphism and morphological transition of *C. albicans* can potentiate the therapy. The hyphal and filamentous morphologies are completely repressed by piperine which affirm its antivirulent potential. Similar to filamentous morphology, wrinkling in *C. albicans* is contemplated to be more virulent, as wrinkled colonies necessitate hyphal development and adhesins production (Lindsay et al., 2014). Piperine, even at the lowest concentration (8 μg mL^–1^) restricted wrinkling of cells. Preventing the virulent morphologies associated with invasion and biofilm formation substantiates that piperine could possibly thwart invasive candidiasis. Besides preventing virulent morphologies and yeast to hyphal transition, piperine was shown to be proficient in transiting hyphal to yeast cells. Hyphal cells contribute more in the pathogenicity of *C. albicans* infection as mutants that are unable to form hyphae under *in vitro* conditions are generally found to be less virulent (Lo et al., 1997). Transmuting the pathogenic hyphal form to less-virulent yeast state will increase the susceptibility of *C. albicans* cells to host immune response, as well as to therapeutics. Thus, piperine can not only be used for prevention of *C. albicans* infection, but also can act as an effective therapeutic regimen for established biofilm infections.

A common concern for any agent with therapeutic potential against microbes is the development of resistance. Due to widespread drug resistance to commonly used antibiotics, it is discreet to identify bioactive therapeutics that exhibit low resistance development. Also, *Candida* spp., develops several molecular mechanisms to resist attack by antifungal drugs (Krishnasamy et al., 2018). Though potential antibiofilm agents do not induce Darwinian selection pressure on microbes, the ability of the microbes to survive under selective-stressful environmental conditions can result in resistance development. The results of spontaneous resistance assay showed that *C. albicans* did not develop spontaneous resistance to piperine even at the concentrations 10-fold higher than the 2 × BIC. Moreover, this is the first report to study the spontaneous antibiofilm resistance development.

Further, the gene expression analysis revealed that piperine significantly downregulated the expression of biofilm-, hyphal-, and filamentation-specific genes that are in line with the phenotypic assays. *EFG1* (gene encoding for enhanced filamentous growth protein 1) and *CPH1* (gene encoding for transcription factor CPH1) are two major transcription factors that are characterized as morphological regulators and are known to be involved in promoting filamentous growth and regulation of the expression of several genes with crucial function in invasion and/or biofilm formation (Dieterich et al., 2002; Staniszewska et al., 2015). Piperine treatment to *C. albicans* significantly downregulated the expression of both the transcription factors *EFG1* and *CPH1* by 5.7- and 4.5-fold, respectively. Another key filament-specific transcriptional regulator, *UME6*, which also regulates the transition of yeast to hyphae form (Banerjee et al., 2013), was found to be 2.1-fold downregulated by piperine treatment. *ECE1*, a hyphal specific gene, was found to be downregulated by 1.5-fold. *HWP1*, a gene that encodes for hyphal wall protein 1, is a major hyphal cell wall protein that plays a role of adhesin and is also required for hyphal development, mating, maintenance of biofilm integrity, attachment to host, etc. Moreover, *hwp1* mutant *C. albicans* was shown to be defective in germination, hyphal development, and reduced interaction with the host system *in vivo* system (Tsuchimori et al., 2000). Piperine treatment resulted in 3.7-fold downregulation of *hwp1* which is well correlated with its potential in inhibition of hyphal formation as observed in phenotypic assays. Adhesin protein-encoding genes *EAP1* (cell wall adhesin) and *ALS3* and *ALS1* (agglutinin-like proteins) are involved in the process of adhesion to host surfaces and biofilm formation both *in vitro* and *in vivo* conditions (Hoyer, 2001; Li and Palecek, 2003). Piperine treatment significantly diminished the expression of both the adhesin genes *EAP1* and *ALS3* and *ALS1* by 1. 8-, 5-, and 1.8-fold, respectively, which is directly corroborated with the decreased biofilm formation *in vitro* and reduced colonization in the *in vivo C. elegans* infection model. Furthermore, *RAS1*, which is involved in morphogenetic switching of *C. albicans* from yeast to polarized filamentous form (Martin et al., 2005) was found to be downregulated by 1.2-fold, due to the activity of piperine. This result further substantiates the potential of piperine to inhibit yeast to hyphal transition. *HST7*, which encodes for a serine/threonine-protein kinase STE7 homolog protein that plays a crucial role in mating (Chen et al., 2002), was found to be 5.5-fold less expressed in piperine-treated *C. albicans.* In addition, downregulation of *CDR2* and *CDR4*, a multidrug transporter gene (Mukherjee et al., 2003), signifies that piperine-treated *C. albicans* cells are less probable to develop antibiotic resistance. *TUP1*, a negative regulator of hyphal and filamentation related genes is found to be upregulated which further substantiates the antihyphal potential of piperine (Goffena et al., 2018).

For a bioactive compound to be taken for clinical applications, it should be non-toxic and must be effective *in vivo* conditions. The *in vivo* toxicity and antibiofilm potential of piperine was evaluated by using a simple eukaryotic model organism, *C. elegans*, which is the widely accepted model organism for toxicological analysis, as it shares homology with 83% of human genes (Dengg and van Meel, 2004; Henricson et al., 2004). The fact that fundamental biological processes are highly conserved between *C. elegans* and humans, application of *C. elegans* in biomedical research and drug discovery process is constantly expanding. Furthermore, *C. elegans* was considered in the drug discovery process at the preclinical stage due to numerous advantages including morphology, lifestyle, growth, brood size, molecular and genetic amenabilities, etc. Due to these several advantages, it has been suggested that *C. elegans* could be incorporated in the preliminary drug discovery and target identification process, as well as in the secondary level, for toxicity screening and preclinical validation of drugs (Arya et al., 2010; Manoharan et al., 2017; Lee et al., 2018). *C. elegans* toxicity data can provide information about the drug effect on digestive, reproductive, endocrine, sensory, and neuromuscular systems. Moreover, research intended to rank toxicity in *C. elegans* have consistently shown a good correlation with rodent oral LD50 ranking (Hunt, 2017). Apart from involvement in toxicological analysis, *C. elegans* is being used as a model host system to review mammalian virulence and pathogenesis of bacterial and fungal organisms including *C. albicans* (Breger et al., 2007; Okoli et al., 2009). The *in vivo* study revealed that piperine does not display a lethal effect to *C*. *elegans.* Moreover, better survival was observed in piperine-treated *C. albicans* infection group than the infection control which clearly depicts that piperine assisted *C. elegans* to combat *C. albicans* infection. Better survival in *C. albicans*-infected worms was observed in a concentration-dependent manner.

Piperine, the bioactive natural molecule that belongs to the *Piperaceae* family, can be detected as the major component in piper species such as *Piper nigrum* L. (black pepper), *Piper longum* L. (long pepper). Additionally, piperine can also be found in the leaves of *Rhdodendron faurie*, seeds of *Anethum sowa*, *Fructus piperis* Long, and bark of *Careya arborea.* Piperine belongs to the largest class of secondary metabolites, alkaloids, and it imparts the pungent taste to pepper that is responsible for its application in human dietary utilization and as a food ingredient for centuries. In addition to dietic application, piperine has been used in various traditional systems of medicine for curing array of disorders such as rheumatism, flu, muscular disorder, cold, fever, etc., and for increasing circulation of blood, flow of saliva, and stimulation of appetite and peristalsis. Equal proportion of black pepper, long pepper, and rhizomes of ginger are used for the formulation of Trikatu, an important ancient formulation that is still in use as an aid for digestive ailment. It has been reported that the effect of both black and long pepper is primarily caused by the presence of piperine (but not exclusively) (Meghwal and Goswami, 2013). A composition comprising a minimum of 98% of pure alkaloid piperine has been patented for its application as a bioavailability enhancer and augmentation of gastrointestinal absorption and systemic utilization of nutrients and nutritional supplements (patent no. US5536506A). Numerous reports are available on the potential of piperine to enhance the serum levels of drugs and nutrients in animals and human beings which includes drugs such as vasicine, rifampicin, theophylline, phenytoin, pyrazinamide, isoniazid, and propranolol and nutrients such as fat-soluble beta-carotene, water soluble vitamin B6, and minerals. Selvendiran et al. (2003) have reported that there is either no significant change in the health status nor toxicity in the Swiss albino mice treated with piperine when compared to control animals. Similarly, several other animal studies through oral administration have evaluated the various potentials of piperine (Lee et al., 1984; Suresh and Srinivasan, 2010; Johnson et al., 2011). In a human clinical study, oral administration of piperine has been shown to increase the swallow response of patients with oropharyngeal dysphagia (Rofes et al., 2014). Randomized clinical trials involving humans subjected to oral administration of piperine have been reported for the treatment of diverse diseases, and none of them have stated that piperine is toxic to neither animals nor humans (Badmaev et al., 2000; Volak et al., 2013; Panahi et al., 2017). Piperine has been evaluated for its immunomodulatory effect and reported to increase the total WBC count, bone marrow cellularity, etc. (Sunila and Kuttan, 2004; Pathak and Khandelwal, 2009). Owing to its numerous health benefits, traditional usage, generous clinical records, innocuous nature, and being expended as a therapeutic molecule for treatment of certain diseases, piperine could be considered as a risk-free candidate molecule for dentifrice application. Moreover, one potent nature of piperine that makes it as a desirable drug candidate for treating oral infectious diseases lies in its ability to increase the salivary flow rate. One of the predisposing host factors associated with oral candidiasis includes the reduced salivary flow rate (Radfar et al., 2003; Williams et al., 2011; Al-Kebsi et al., 2018). Along with the antibiofilm and antihyphal activity, increasing the salivary flow rate is an added value to the therapeutic potential of piperine for oral candidiasis.

Piperine treatment was also found to be safe to the HBECs. Hence, together with these results and reports, piperine can be considered as safe for clinical purpose in human system for treating biofilm-associated *C. albicans* infections.

Altogether, the results have demonstrated the antibiofilm and antihyphal potential of a plant alkaloid molecule, piperine. The proficiency of piperine to inhibit morphological transitions and phenotype switching, with low potential for resistance development, exemplifies its therapeutic potential. Additionally, its non-toxic nature with respect to HBECs and *in vivo* antiinfective efficacy marks piperine as a promising drug molecule for preventing as well as treating the biofilm-associated *C. albicans* infection, specifically oral candidiasis.

## Data Availability Statement

All datasets generated for this study are included in the manuscript.

## Ethics Statement

The studies involving human participants were reviewed and approved by Institutional Ethics Committee, Alagappa University (IEC Ref No: IEC/AU/2018/5). The patients or participants provided their written informed consent to participate in this study.

## Author Contributions

Both authors designed the study, read and approved the final version of the manuscript. AP performed the experiments, analyzed the data, prepared the figures and tables, and wrote the manuscript. SP revised the manuscript.

## Conflict of Interest

The authors declare that the research was conducted in the absence of any commercial or financial relationships that could be construed as a potential conflict of interest.
